# The evolving role of physical therapists in the long-term management of chronic low back pain: longitudinal care using assisted self-management strategies

**DOI:** 10.1590/bjpt-rbf.2014.0180

**Published:** 2016-06-30

**Authors:** Paul F. Beattie, Sheri P. Silfies, Max Jordon

**Affiliations:** 1 Doctoral Program in Physical Therapy, Department of Exercise Science, Arnold School of Public Health, University of South Carolina, Columbia, SC, USA; 2 Department of Physical Therapy & Rehabilitation Sciences, Drexel University, Philadelphia, PA, USA; 3 Physical Therapist, Mobility Research Clinic, Richland-Palmetto Health, Columbia, SC, USA

**Keywords:** rehabilitation, spinal disorders, chronic pain

## Abstract

**Background:**

Longitudinal studies have shown that the symptoms of chronic low back pain (CLBP) will follow an episodic trajectory characterized by periods of high and low pain intensity that can persist for many years. There is a growing belief that the contemporary approach of limiting physical therapy to short, but intense courses of treatment for (CLBP) may be sub-optimal because these limited “windows” of clinical care are not congruent with the natural history of this condition. Recent research has suggested that people with CLBP undergo substantial, and individualized long-term variations in the neural processing of nociception over time. This has led to the concept of a “unique biosignature of pain” that may explain much of the variation in a person’s clinical picture. These and other findings have led to the reconceptualization of CLBP as an individualized, and continually evolving condition that may be more suitably managed by empowering the patient toward self-management strategies that can be modified as needed over time by the PT.

**Objectives:**

The purpose of this Master Class Paper is to describe an emerging approach for the treatment of CLBP that emphasizes the formation of a long-term therapeutic alliance between the patient and the PT with an emphasis on individualized, patient-preferred approaches for activity-based self-management as an alternative to the contemporary approach of short, intense episodes of care directed toward pain reduction.

**Conclusion:**

Longitudinal care using assisted self-management strategies is more congruent with the natural history of CLBP than are traditional approaches for PT intervention. This approach may empower patients to undergo lifestyle changes that will favorably influence long-term outcomes; however additional research is needed.

## BULLET POINTS

• Chronic low back pain (CLBP) is even more complicated than previously believed.
• This condition is usually associated with substantial loss of muscle function and is often related to mal-adaptive changes in the way patients perceive and appraise pain.
• These and other factors help to explain why CLBP typically has a long-term trajectory that is not strongly impacted by traditional physical therapy (PT) approaches that utilize brief, intense episodes of treatment.
• Longitudinal care using assisted self-management strategies addresses the long-term trajectory of CLBP by emphasizing the formation of an ongoing therapeutic alliance between the patient and the PT that incorporates individualized, patient-preferred approaches for activity-based self-management.
• This approach may provide the long-term stimuli needed to address the muscular and neurologic impairments associated with CLBP thus making it a useful alternative to the traditional approach of short but intense episodes of care, however additional research is needed.

## Introduction: is it time for a new model of delivery for back pain?

Chronic low back pain (CLBP) continues to be the most prevalent cause of disability and lost work time among working-age adults in industrialized countries^1^ ^-^ ^5^. Despite an enormous growth in the research evidence base, the worldwide prevalence of CLBP is not decreasing and may actually be increasing in recent years^1^ ^,^ ^3^ ^,^ ^4^. The illness burden that is associated with this growing prevalence of CLBP has had substantial impact on all stakeholders and has prompted a call for a reassessment of traditional treatment approaches^6^ ^-^ ^8^. This call creates a challenge and an opportunity for physical therapists. For example, surgical and pharmacologic interventions for CLBP have been ineffective for many people and often present an elevated risk of adverse events^9^. In contrast to this, the non-invasive, non-pharmacologic interventions used by physical therapists present an attractive alternative. Not surprisingly, patients with CLBP comprise the largest cohort of people who receive physical therapy and this condition represents one of the most highly researched areas in the field^10^. Unfortunately, most high-quality research studies and systematic reviews suggest that although various physical therapy interventions may be of short-term value, they are likely to have limited effect on long-term outcomes^11^ ^-^ ^17^.

There are many potential reasons for the lack of long-term impact of physical therapy interventions for the treatment of people with CLBP. One under-investigated possibility is that the traditional approach of utilizing PT for a short, but intense course of treatment such as the common approach of 12 visits over a 4-8 week period^18^ may be sub-optimal because this does not typically generate the dosage and duration needed to favorably influence tissue changes and promote the behavioral modifications needed to maximize the likelihood of success^6^ ^,^ ^7^ ^,^ ^19^ ^-^ ^27^. The purpose of this Master Class Paper is to describe an emerging approach for the delivery of physical therapy care for CLBP that utilizes longitudinally supported self-management within a therapeutic alliance to develop individualized, patient-preferred approaches for activity-based self-management. This approach is conceptualized as an alternative to the contemporary approach of short but intense episodes of care directed toward pain reduction.

## The reconceptualization of back pain as a chronic, but manageable condition

### Epidemiologic studies suggest that the natural history of LBP is much more complex than previously believed

Patients with low back pain have traditionally been divided into 2 major groups: acute and chronic^28^ ^,^ ^29^. Acute LBP has alternatively been used describe the initial 6 weeks to 3 months following the first occurrence of low back pain, or this same time period following a flare-up of symptoms that had previously gone away. The classification of chronic low back pain (CLBP) has been used to describe patients whose symptoms have persisted past the point of likely tissue healing, usually greater than 3-6 months. CLBP may be further sub-divided based upon severity of symptoms and impact upon one’s life, i.e. there is a large “bandwidth” that ranges from individuals who have annoyance-level pain to those with severe loss of function and disability^8^. Several well-conducted, prospective cohort studies suggest that the majority (~80%) of those people with acute LBP will have recovery in 6 to 8 weeks and may not need definitive treatment; while those people who have had symptoms for more than 3-6 months (CLBP) have been generally believed to have a much lower likelihood of recovery and may present with more complex treatment challenges^30^ ^-^ ^34^. The differences, however, in the natural history between acute and chronic LBP may be much more complex than that. For example, Henschke et al.^35^ enrolled 973 patients of working-age with acute LBP at the time of symptom onset (an inception cohort design) and followed them over time. Fifty percent of patients had returned to work within 2 weeks, while 83% had returned within 3 months supporting the belief that most people with recent onset of symptoms get better in a short period of time. However, 28% of patients still reported symptoms at 12 months following onset suggesting that acute LBP may not have as favorable a prognosis for recovery as suggested in the previous estimates. Other researchers have reported unexpectedly high recurrence rates^36^ ^-^ ^39^ in people with acute LBP supporting the premise that, for a considerable number of people, acute LBP probably represents a naturally recurring condition characterized by periodic “flare-ups” of symptoms.

An illustration of this is described by Costa et al.^36^ who performed a meta-analysis of inception-cohort studies that had investigated the clinical course of people with acute LBP. These authors reported that while most people showed marked recovery in pain within the first 6 weeks after symptom-onset, rates of recovery slowed after that and most individuals still had noticeable symptoms at 1 year.

### Exciting new imaging research may provide biologic reasons why the natural history is so complex

Advances in imaging of the lumbar spine and central nervous system are creating a new understanding of the mechanisms by which the symptoms of LBP are propagated, appraised and influenced by both intrinsic and extrinsic factors^40^ ^-^ ^50^. Although these findings are preliminary, it is apparent that unique and very individualized variations in the structure and function of key neural and musculoskeletal tissues are highly linked to chronic LBP^40^ ^,^ ^50^ ^-^ ^58^. For example, maladaptive changes in the gray matter of the brain, sometimes referred to as “neuroplastic changes” are frequently observed in people with CLBP^40^ ^,^ ^43^ ^,^ ^53^. Some authors have proposed that these changes may also be linked to biobehavioral factors that can be associated with adverse pain processing^40^ ^,^ ^43^ ^,^ ^51^. It has been proposed, but not yet clearly demonstrated, that these changes are reversible however this may require substantial, repetitive stimuli over time^59^ ^,^ ^60^.

In addition to their potential to alter the appraisal of sensory information, neuroplastic changes may also contribute to difficulty with the planning and implementation of motor tasks^23^ ^,^ ^47^ ^,^ ^49^ ^,^ ^61^ ^,^ ^62^. This problem could be a key barrier when teaching patients spinal exercises, e.g. people with CLBP may require a substantial number of repetitions over time before the exercises can be performed correctly. This can be conceptualized as providing a long-term stimulus to “reset” the nervous system.

The overall impact of these neuroplastic changes has led to the concept of a “unique or personalized biosignature of pain” that may explain much of the variation in a person’s clinical picture over time^27^ ^,^ ^45^ ^,^ ^53^ ^,^ ^63^ ^,^ ^64^. Wide variations in the central processing of pain between different people may explain, in part, the limited success of treatment-based clinical prediction rules that primarily rely upon physical examination findings that may not be strongly related to an individual’s overall “pain experience”^64^ ^-^ ^66^.

Another interesting, recent finding is that spinal muscles may undergo more substantial morphologic and neurologic changes in response to injury than was previously believed. Beneck and Kulig^19^ reported an unexpected high loss of volume in the lumbar multifidus at the L5-S1 even in high functioning individuals with CLBP^19^. Other investigators have reported similar findings and have observed substantial fatty infiltration occurring within the back muscles of people with CLBP^20^ ^,^ ^21^ ^,^ ^67^. The impact of these findings is unknown but they may be linked to pain due to an increase in the size of the afferent receptor field and the decreased influence of mechanoreceptors. This may lead to an increase in the activation time of muscles and contribute to a delay in cognitive processing that could make it difficult to learn the performance of therapeutic exercises^62^ ^,^ ^68^.

Although the exact cause is unknown, this change in muscle morphology may be associated with degenerative disc disease and may be the result of nerve compression, muscle trauma, instability and/or latent infections^69^. It is unclear if these changes can be reversed by exercise, but basic concepts of muscle biology suggest that a long term stimulus is likely to be needed to overcome atrophy of spinal muscles and to regain proper muscle morphology and function. This may be more complicated than simply prescribing exercises. For example, Hodges and colleagues^23^ have demonstrated linkages between structural impairment of peripheral tissues and deficits in motor control. This may explain some of the lack of effectiveness of motor control exercises^70^.

### Advances in biobehavioral research also help to explain the complex natural history of LBP

A substantive body of literature has identified key factors such as excessive pain catastrophizing, elevated fear avoidance beliefs, perceived injustice, and somatization that are likely manifestations of maladaptive pain beliefs^71^ ^-^ ^80^. The importance of identifying and addressing these factors is currently being investigated in the TARGET trial being performed in the United States that uses the STartT Back questionnaire^81^ and other measures. Although no results from this study are yet available, it is well agreed that individuals who present with elevated biobehavioral factors may need additional, and possibly ongoing, types of cognitive interventions, such as pain neuroscience education^59^ to develop a “healthy appraisal of pain” that would facilitate their ability to participate in therapeutic levels of physical activity.

Based upon this work, arguments can be made that back pain represents a persistent, episodic disorder that is not likely to spontaneously resolve in many people. This observation may partially explain why so many intervention studies for acute LBP show good short-term benefit but demonstrate very little long-term benefit. Conceptually, for many people back pain is a “moving target” that may require considerable recovery time. Considering this, arguments can be made that intervention approaches that effectively involve the patient in long-term performance of therapeutic types of physical activity are likely to be a potentially valuable alternative to current delivery models. These approaches seek to empower the patient toward long-term self-management by emphasizing patient-preferred physical activities that can therapeutically target injured tissues and be modified as needed over time by the physical therapist. The key challenge for physical therapists is to develop and validate the intervention “package” and delivery-model that can provide patients with the best opportunity for a favorable outcome.

## Limitations with the traditional approaches for delivering care for back pain

In many community-based or specialty care physical therapy facilities people seeking treatment for CLBP are likely to receive an “episode of care” that often lasts 1-2 months with frequent visits^18^. The emphasis of treatment typically is targeted toward pain control with the addition of a “home program” at or near the end of care. This approach has the advantage of allowing a concentrated application of treatment and is popular with clinic administrators and third party payers because it allows a specific billing period. Not surprisingly, a large number of clinical investigations that assess PT intervention for CLBP assess treatments that are administered in this same time period.

These limited “windows” of clinical care are however not congruent with the natural history of CBLP. Longitudinal studies have shown that the symptoms of CLBP will follow an episodic trajectory characterized by periods of high and low pain that can persist for many years^35^ ^-^ ^39^. In addition these concentrated, short duration treatment approaches may not be effective for empowering patients to perform adequate physical activity and exercise to manage symptoms over time^6^ ^,^ ^26^ ^,^ ^81^ ^,^ ^82^. Importantly, recurrence of symptoms may be viewed as failure of treatment and have detrimental consequences^75^ ^,^ ^83^.

## Self-management as a treatment approach for chronic illness

Self-management of chronic diseases is an emerging concept that is typically based upon social-cognitive and health beliefs theories that seek to empower the patient to use exercise and healthy lifestyle approaches to cope with his or her condition^84^ ^-^ ^88^. Interestingly, preliminary clinical investigations of self-management approaches for arthritis, back pain and diabetes have demonstrated only modest success^87^ ^-^ ^91^. One likely explanation for this limited outcome is in the way they have been administered. These programs have traditionally used lengthy initial periods of education that are often done in a group format followed by instruction to continue the self-management interventions without additional consultation^92^. Potential flaws in this model are that the group approach to instruction fails to develop a meaningful therapeutic alliance in which the patient and provider work together to develop a patient-preferred approach^93^ ^-^ ^95^, and it does not allow for the careful monitoring of progress, constructive feedback/encouragement, and periodic adjustments to the self-management program.

## Longitudinally Supported Self-Management and the therapeutic alliance

Longitudinally Supported Self-Management (LSSM) acts to interface theories of self-management of chronic disease with a support system keyed by a strong therapeutic alliance between the patient and the PT. This alliance allows the patient to have ongoing, additional consultations after a brief initial course of individualized treatment. The PT becomes the primary provider who will monitor for red and yellow flags while developing and implementing long-term management strategies. These strategies are designed to maximize compliance based upon patient empowerment and patient-preferred physical activity (Table 1)^96^. The key component of LSSM is the patient-therapist interaction, ie, “the Therapeutic Alliance”^94^ ^,^ ^95^. Numerous studies have demonstrated that this interaction is the strongest predictor of patient satisfaction with physical therapy care, and may be a key contributor to successful outcome^93^ ^,^ ^96^ ^-^ ^99^. In 2014, an innovative study by Fuentes et al.^95^ reported that individuals with chronic LBP who received interferential electrical stimulation to the lumbar erector spinae muscles, that was combined with a nurturing “therapeutic alliance” between the patient and therapist, had significantly better outcomes than did those subjects who received the same dosage of interferential current with minimal interaction with the practitioner. The influence of this alliance may be conceptualized by some as illustrating that an ineffective or “placebo” effect occurred during the treatment, which suggests a negative connotation that infers the intervention is “fake”^100^. A growing body of literature however, is demonstrating substantive physiologic events are created by “non-specific” or placebo treatments that, in turn, have an enormous therapeutic effect on pain^101^ ^-^ ^104^. It can be conceptualized that impact of the therapeutic alliance is added to the treatment response to determine the overall value/impact of the treatment. This observation suggests that the PT’s interaction is a key therapeutic agent. For example, it may be that how the treatment is delivered or presented to the patient is the critical factor to promote adherence. Maintaining longitudinal continuity, i.e. having the same therapist treat a patient over his course of care, will potentially enhance the therapeutic alliance and patient satisfaction with care^93^.

**Table 1:** A list of challenges faced by people with chronic low back pain and the processes by which they are address with longitudinally supported self-management.

**Challenge**	**Process**
Mal-adaptive pain beliefs	• Pain science education using patient-preferred learning approaches
Need to “de-medicalize” the condition	• Emphasis on patient empowerment and strategies to maximize self-efficacy
A large dose of active participation over a long time period is required to addresses neuroplasticity and muscle atrophy	• Emphasis on exercise-based lifestyle • Patient-preferred activities to maximize compliance • Awareness of time required for healing
Exacerbations of pain are likely to occur	• Conceptualize exacerbations of pain as normal and non-threatening • Provide the availability of “first aid” or “tune-up” visits
Need for support system while also emphasizing independence	• Therapeutic alliance and longitudinal continuity of care

### Goals of the initial treatment period

**Figure 1:**
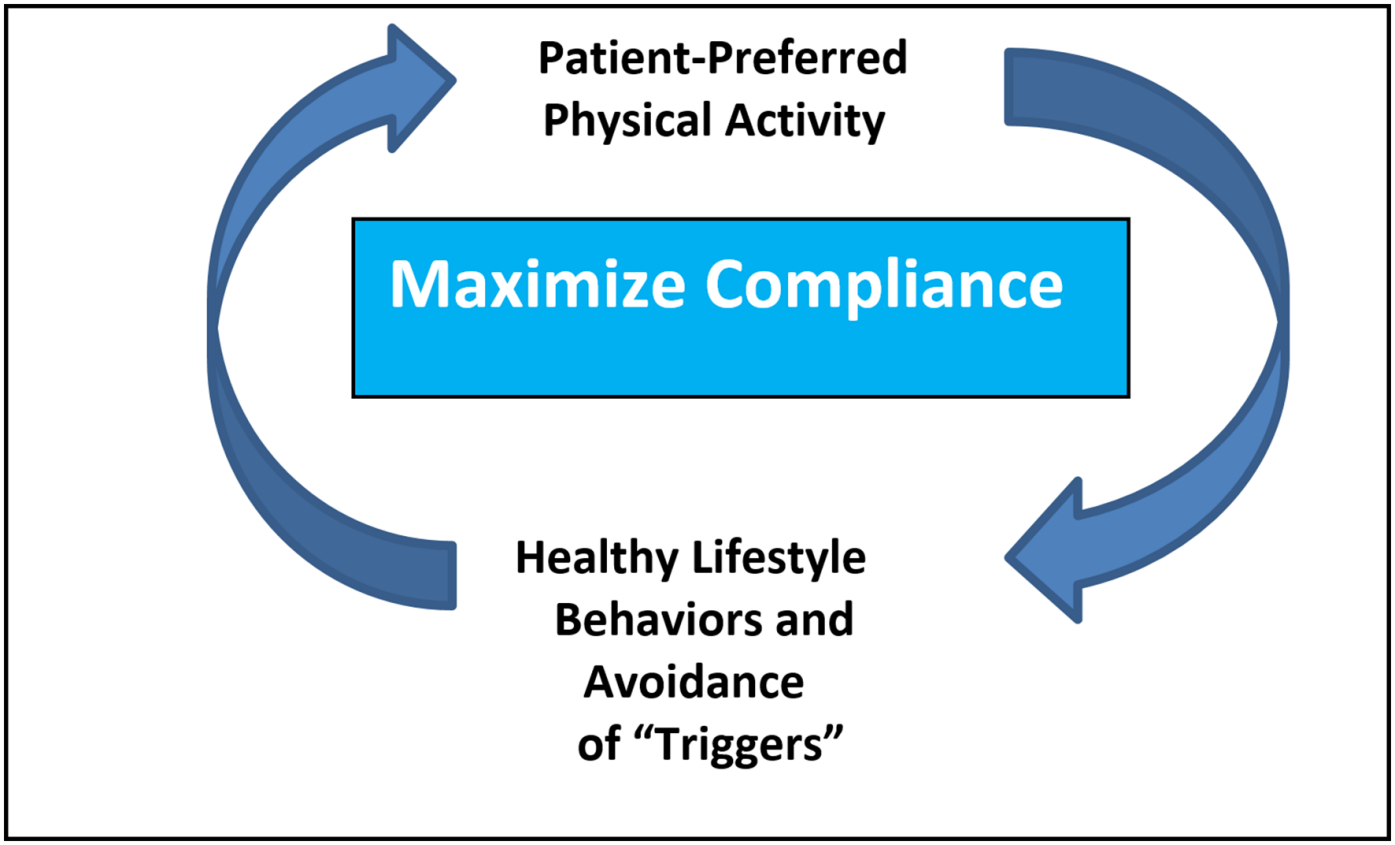
Strategies for longitudinally supported self-management are focused upon patient-preferred activities to maximize the likelihood of long-term compliance.

The proposed approach for treatment administration using longitudinal supported self-management begins with brief series of 2-4 visits in a 1-2 week period for in depth examination that includes identification of “red, yellow and blue flags”^105^ ^,^ ^106^. This examination includes an accurate medical and biobehavioral screening using appropriate measures such as the STarTBack assessment^81^. During this time the patient and PT work closely to determine if the patient’s goals, attitudes, and beliefs are consistent with self-management^107^ ^,^ ^108^. The emphasis of the treatment approach is focused upon the long-term management of symptoms through positive ideation of pain, reduction of modifiable triggers (Table 2)^109^ and maximization of loading tolerance using patient-preferred physical activities (Figure 1). If successful, this approach dovetails with the “healthy lifestyle” approach that has had positive effects on many other conditions^24^ and will become an important selling point to all stakeholders. The initial treatment emphasizes education regarding pain theory (consistent with Modern Neuroscience Approach)^59^ to which there are several approaches that PTs should consider based upon the patient’s preference for learning. The initial key short term objective is to reduce and manage symptom-triggers while promoting enhancers such as strategies to stabilize the spine during unanticipated loads and ways to minimize chronic end-range loading. The second objective is to develop a “healthy” appraisal of pain symptoms. This is based upon a patient conceptualization of LBP as chronic condition whose episodic symptoms and impact can be self-managed by appropriate physical activity (Table 1). The overall intervention strategy is based upon an active approach, for example, Blyth et al.^89^ reported that self-management strategies using passive approaches (medication, hot packs) increased the likelihood of disability (adjusted OR=2.59) while active strategies such as exercise decreased the likelihood (adjusted OR=.2). Central to this finding is the understanding that this process takes time and effort. It is important to note that a limited amount of pain control interventions using passive treatments such as manual therapy or dry needling may be valuable for short periods during exacerbations but they must not be conceptualized as a the primary focus of treatment.

**Table 2:** Likely physical or psychosocial “triggers” of an episode of low back pain (N=999). From a case-crossover study by Steffens et al.^109^ (permission applied for).

**Triggers**	**Odds Ratio (95% CI)**
**Physical Factors** Manual Tasks Involving	
Heavy loads	5.0 (3.3-7.4)
Awkward posture	8.0 (5.5-11.8)
Objects not close to the body	6.2 (2.4-15.9)
People or animals	5.8 (2.3-15.0)
Unstable, unbalanced, and/or difficult to grasp	5.1 (2.4-10.9)
Moderate or vigorous physical activity	2.7 (2.0-3.6)
Vigorous physical activity only	3.9 (2.4-6.3)
**Psychosocial Factors**	
Distracted during an activity or task	25.0 (3.4-184.5)
Fatigued/tired	3.7 (2.2-6.3)

The overarching goal is the development of a self-management approach that is based upon patient-preferred physical activities. These activities should include biomechanically sound exercises within the patient’s unique structural and physiologic capacities. These exercises/activities must be patient-preferred and developed to maximize compliance. Regaining adequate strength and motor control will take a long time with adequate dosages. This is likely to take many months and is not likely to be dramatically changed in a short episode of care. This may explain some of the variance in poor outcome from short term studies. Long-term maintenance of muscle strength is an ongoing and life-long process. This is very important for patients to understand.

### Longitudinal monitoring

This program is monitored and adjusted over time as the patient’s condition evolves and involves periodic “rechecks” or tune-ups. There is lots of room for creativity relative to the type of activity and the method of communication with the patient. “Flare-ups” will occur but these are conceptualized as a normal part of recovery. The advantage is that the patient is “in the system” and has a strong therapeutic alliance with his/her PT that allows for additional treatment for pain control when needed. Knowing this will reduce the patient’s anxiety and fear allowing further patient empowerment.

### The potential advantages of Longitudinally Supported Self-Management and the therapeutic alliance

A major advantage of LSSM is that it can allow considerable time for a favorable exercise effect on spinal tissues such as increases in muscle strength, joint mobility, disc diffusion, bone density, and the possible reversal of unfavorable neuroplastic adaptations. This effect is likely to take months and require on-going maintenance and is virtually never obtained in contemporary treatment approaches. By emphasizing self-management from the beginning of treatment patients become less reliant upon the medical system to “cure” their problem. Waddell has referred to this as “demedicalization”^8^.

Adherence with exercise programs is a major concern^110^. Patient preference may increase long-term adherence. LSSM allows patients to identify and try-out exercise approaches that they prefer; for example, emerging evidence suggests that performance of yoga^111^ a or Pilates^112^ ^,^ ^113^ exercises has evidence of effectiveness and be a more attractive option for patients than conventional exercises.

## Limitations and challenges

Successful LSSM will face several challenges. This approach will not be appropriate for everyone with CLBP. Patients who are averse to exercise or have low degrees of self-efficacy regarding self-management would potentially be less likely to have a successful outcome^78^. In addition, LSSM places new challenges on the PT to develop and maintain a strong, longitudinal therapeutic alliance^83^ ^,^ ^114^ ^-^ ^116^. This task may require the use of telemedicine and other remote technologies that would necessitate the development of new models for treatment billing.

The current body of literature that has examined self-management strategies for the treatment of chronic musculoskeletal conditions suggests that these approaches have had limited benefit on outcomes^84^ ^-^ ^92^. LSSM is likely to improve these outcomes by the use of improved patient monitoring, a strong support system through the therapeutic alliance, and an emphasis on patient-preferred physical activity; however, high quality studies need to be performed to identify the characteristics of likely responders and to determine the overall cost-effectiveness of this approach when compared to traditional delivery models.

## Summary

Longitudinally supported self-management (LSSM) for CLBP seeks to reduce maladaptive pain behaviors by using patient-selected physical activities as a potential way to target unique neurologic and muscular impairments associated with this condition. LSSM is based upon shared decision-making that arises from a strong therapeutic alliance between the patient and PT. There is biologic support for this approach but clinical studies are needed to determine the impact of this approach.
